# The role of bioreductive activation of doxorubicin in cytotoxic activity against leukaemia HL60-sensitive cell line and its multidrug-resistant sublines

**DOI:** 10.1038/sj.bjc.6602639

**Published:** 2005-06-07

**Authors:** D Kostrzewa-Nowak, M J I Paine, C R Wolf, J Tarasiuk

**Affiliations:** 1 Department of Biochemistry, University of Szczecin, 3a Felczaka St, 71-412 Szczecin, Poland; 2 Cancer Research UK Molecular Pharmacology Unit, Biomedical Research Centre, Ninewells Hospital and Medical School, Dundee DD1 9SY, UK

**Keywords:** doxorubicin, NADPH cytochrome *P*450 reductase, redox cycling, reactive metabolite(s), HL60 human promyelocytic leukaemia, multidrug resistance

## Abstract

Clinical usefulness of doxorubicin (DOX) is limited by the occurrence of multidrug resistance (MDR) associated with the presence of membrane transporters (e.g. P-glycoprotein, MRP1) responsible for the active efflux of drugs out of resistant cells. Doxorubicin is a well-known bioreductive antitumour drug. Its ability to undergo a one-electron reduction by cellular oxidoreductases is related to the formation of an unstable semiquionone radical and followed by the production of reactive oxygen species. There is an increasing body of evidence that the activation of bioreductive drugs could result in the alkylation or crosslinking binding of DNA and lead to the significant increase in the cytotoxic activity against tumour cells. The aim of this study was to examine the role of reductive activation of DOX by the human liver NADPH cytochrome *P*450 reductase (CPR) in increasing its cytotoxic activity especially in regard to MDR tumour cells. It has been evidenced that, upon CPR catalysis, DOX underwent only the redox cycling (at low NADPH concentration) or a multistage chemical transformation (at high NADPH concentration). It was also found, using superoxide dismutase (SOD), that the first stage undergoing reductive activation according to the mechanism of the redox cycling had the key importance for the metabolic conversion of DOX. In the second part of this work, the ability of DOX to inhibit the growth of human promyelocytic-sensitive leukaemia HL60 cell line as well as its MDR sublines exhibiting two different phenotypes of MDR related to the overexpression of P-glycoprotein (HL60/VINC) or MRP1 (HL60/DOX) was studied in the presence of exogenously added CPR. Our assays showed that the presence of CPR catalysing only the redox cycling of DOX had no effect in increasing its cytotoxicity against sensitive and MDR tumour cells. In contrast, an important increase in cytotoxic activity of DOX after its reductive conversion by CPR was observed against HL60 as well as HL60/VINC and HL60/DOX cells.

The anthracycline antitumour agent, doxorubicin (DOX), is one of the most effective drugs, currently available for the treatment of various human neoplastic diseases including leukaemias, lymphomas, sarcomas, carcinomas and breast cancers (Wiernik and Dutcher, 1992; Lown, 1993). However, their clinical usefulness is limited by the occurrence of multidrug resistance (MDR) associated with the presence of membrane transporters (e.g. P-glycoprotein, MRP1), belonging to the ATP-binding cassette protein family (Gottesman and Pastan, 1993; Germann, 1996; Marie *et al*, 1996; Borst *et al*, 2000). These transporters are responsible for the active ATP-dependent efflux of drugs out of resistant cells resulting in the decreased intracellular accumulation insufficient to inhibit resistant cell proliferation (Zaman *et al*, 1994; Paul *et al*, 1996).

Different mechanisms have been proposed for anthracycline antitumour effects including DNA intercalation with consequent inhibition of DNA biosynthesis, free radical formation with induction of DNA damage, alkylation of DNA and DNA crosslinking, inhibition of topoisomerase II, activation of signalling pathways and apoptosis (for a recent review, see Gewirtz, 1999). Free radical generation by DOX is linked to its one-electron reduction by cellular oxidoreductases. This process comprises the one-electron transfer from reduced nucleotides, which converts the anthracycline molecule to a semiquinone radical form DOX^•^. The process of a one-electron transfer is catalysed by a range of cellular oxidoreductases, mainly NADH dehydrogenase, NADPH cytochrome *P*450 reductase (CPR), xanthine oxidase (Mordente *et al*, 2001; Pawłowska *et al*, 2003) and nitric oxide synthase (Vasquez-Vivar *et al*, 1997; Garner *et al*, 1999). Subsequent nonenzymatic semiquinone radical reoxidation by molecular oxygen can form reactive oxygen species (O_2_^•^, ^•^OH, H_2_O_2_, ^1^O_2_) that interact with various cellular macromolecules, for example, DNA (Begleiter and Leith, 1990), lipids and other cell constituents (Shinha, 1989). Doxorubicin-induced generation of reactive oxygen species was proposed to be a major mechanism of its dose-dependent cardiotoxicity (Lee *et al*, 1991; Havlin, 1992; Gille and Nohl, 1997). It is generally viewed that the reductive metabolism of DOX could also lead to the deglycosylation of the drug catalysed by cellular oxidoreductases (mentioned above) via a one-electron transfer or to the formation of doxorubicinol in the presence of specific enzymes namely aldo–keto reductases and carbonyl reductases catalysing a two-electron reduction of the drug (Minotti *et al*, 1995). These metabolic pathways of DOX play an important role in the detoxication steps (Bernardini *et al*, 1991; Ferrazzi *et al*, 1991), although doxorubicinol can also contribute to the development of the dose-limiting cardiotoxicity (Olson *et al*, 1988; Forrest *et al*, 2000).

On the other hand, there is an increasing body of evidence that the reductive activation of antitumour drugs, for example, mitomycin C, tirapazamine and indoloquinone, could result in covalent binding to DNA (McGuinness *et al*, 1991; Adams and Stratford, 1994; Bailey *et al*, 2001) and lead to the important increase in the cytotoxic activity of these compounds against tumour cells (Patterson *et al*, 1995, 1997; Chinje *et al*, 1999; Cowen *et al*, 2003). Many investigations are also focused on the bioreductive activation of DOX (Bartoszek and Wolf, 1992; Taatjes *et al*, 1997; Bartoszek, 2002). It was evidenced that, in the presence of reductive agents or oxidoreductases, DOX forms reactive intermediates capable of alkylation or crosslinking binding with DNA (Cummings *et al*, 1991; Cullinane *et al*, 1994; Skladanowski and Konopa, 1994). Doxorubicin adducts with cellular DNA have been shown to be formed from reduction of the drug *in vitro* and to involve the C7 position of the reductively activated quinone methide and guanine-base position of DNA (Cullinane *et al*, 1994). It was also evidenced that the covalent linkage of the drug to one of the DNA strand increases remarkably the stability of the duplex in comparison with the complex with noncovalently bound DOX (Zeman *et al*, 1998). The relevance of reductive activation of DOX by CPR for increasing cytotoxic activity of this drug towards sensitive human breast cell line MCF-7 has been also demonstrated using purified rat CPR (Bartoszek and Wolf, 1992). It was also evidenced that the transfection of human CPR cDNA into Chinese hamster ovary cells increased the cytotoxic activity of DOX by 1.8–3.3-fold (Sawamura *et al*, 1996). There are also literature data reporting that the overexpression of CPR in VP79 Chinese hamster fibroblasts (Schmalix *et al*, 1996) and in stably transfected clones of MDA231 human breast cancer cells (Ramji *et al*, 2003) did not confer enhanced sensitivity of these cells to the cytotoxicity of DOX. Additionally, because it is proposed that CPR could also play an important role in the detoxication pathway via the reductive deglycosylation of DOX, some studies were focused on studying the cytotoxic activity against tumour cells presenting decreased CPR expression. It was shown that the suppression of CPR activity in human non-small cell lung carcinoma EBC-1 and PC9 and BALM3 lymphoma cell lines realized via pharmacological methods as well as the antisense approach resulted in increasing cytotoxic activity of DOX (Niitsu *et al*, 2000).

The objective of this study was to examine the role of reductive activation of DOX by the human liver CPR in increasing its cytotoxic activity especially in regard to MDR tumour cells. For this purpose, the ability of DOX to inhibit the growth of human promyelocytic leukaemia HL60 cell line as well as its MDR sublines exhibiting two different phenotypes of MDR related to the overexpression of P-glycoprotein (HL60/VINC) or MRP1 (HL60/DOX) was investigated in the presence of exogenously added CPR and NADPH. It was found that upon CPR catalysis, DOX underwent only the redox cycling (at low NADPH concentration) or the metabolic conversion (at high NADPH concentration). Our assays showed that the presence of CPR catalysing only the redox cycling of DOX had no effect in increasing its cytotoxicity against sensitive and MDR tumour cells. In contrast, an important increase in the cytotoxic activity of DOX after its metabolic conversion by CPR was observed against sensitive HL60 as well as resistant HL60/VINC and HL60/DOX cells.

## MATERIALS AND METHODS

### Reagents

NADPH, superoxide dismutase (SOD) and vincristine were obtained from Sigma Chemical Co. (St Louis, MO, USA). Doxorubicin was kindly provided by Pharmacia-Upjohn (Milano, Italy). CPR from human liver was obtained from C Roland Wolf's Laboratory, Ninewells Hospital and Medical School (Dundee, UK) according to the procedure described earlier (Smith *et al*, 1994).

### Cell culture

HL60 human promyelocytic leukaemia line (Division of Biology, Kansas State University, Manhattan, KS 66506, USA) and its resistant sublines HL60/VINC (overexpressing P-glycoprotein) (McGrath *et al*, 1989) and HL60/DOX (overexpressing MRP1) (Marsh *et al*, 1986; Krishnamachary and Center, 1993) were cultured. The cells were grown in RPMI 1640 (Gibco Limited, Paisley, UK) medium supplemented with 2 mM glutamine and 10% FBS (Gibco Limited, Paisley, UK) at 37°C in a humidified atmosphere of 95% air and 5% CO_2_. HL60/VINC cells were cultured in the presence of 10 nM vincristine and HL60/DOX cells in the presence of 200 nM DOX. All cultures (HL60, HL60/VINC, HL60/DOX) initiated at a density of 10^5^ cells ml^−1^ grew exponentially to about 10^6^ cells ml^−1^ in 72 h. They were counted before the assay using a Burker hemocytometer. Cell viability was assessed by trypan blue exclusion.

### Enzymatic studies

Stock solutions of DOX and NADPH (*C*_0_=10^−3^ M) were prepared just prior to use. Concentrations were determined by diluting stock solutions in water to approximately 50 *μ*M and using an extinction coefficient of *ε*_480_=11 500 M^−1^ cm^−1^ and *ε*_340_=6220 M^−1^ cm^−1^ for DOX and NADPH, respectively.

The reaction mixtures in 0.01 M K_2_HPO_4_/KH_2_PO_4_ buffer (pH 7.25) contained 0–100 *μ*M DOX, 0–2000 *μ*M NADPH, 0–0.1 mg ml^−1^ CPR and 0 or 500 U ml^−1^ SOD. All the reactions were initiated by the addition of CPR and conducted at 37°C. Absorption spectra of DOX were recorded at the indicated time points in the visible region (330–800 nm). NADPH oxidation was measured at *λ*=340 nm using an extinction coefficient of *ε*=6220 M^−1^ cm^−1^. Absorption measurements were made on a Marcel E330 spectrophotometer.

### Cytotoxicity assays

For each cell line, the cytotoxic effects of DOX were determined by incubating cells (10^5^) with 10 different concentrations of the compound for 72 h in standard 48-well plates. The enzymatic samples contained, for DOX acting in redox cycling: 100 *μ*M DOX, 100–350 *μ*M NADPH and 0.1 mg ml^−1^ CPR; for DOX undergoing reductive conversion: 100 *μ*M DOX, 500 *μ*M–2 mM NADPH, 0.1 mg ml^−1^ CPR and 0 or 500 U ml^−1^ SOD (0.01 M K_2_HPO_4_/KH_2_PO_4_ buffer, pH 7.25 at 37°C). The appropriate volumes of the enzymatic sample were added directly to the cell suspensions to yield DOX concentration varying in the ranges of 0.1–100 nM, 5 nM–3 *μ*M and 5 nM–5 *μ*M for HL60, HL60/VINC and HL60/DOX cells, respectively. Control assays were carried out for buffer alone at the highest percentage employed in culture medium (2%, v v^−1^) and for NADPH alone (2, 60 and 100 *μ*M in the case of HL60, HL60/VINC and HL60/DOX cells, respectively) or CPR alone (0.1, 3 and 5 *μ*g ml^−1^ in the case of HL60, HL60/VINC and HL60/DOX cells, respectively) at the highest concentrations used in *in vitro* studies. The values of DOX concentration required to inhibit by 50% (IC_50_) the cell growth were determined by counting the viable cells in the presence of trypan blue using a Burker hemocytometer.

### Statistical analysis

Results are presented as the mean±s.d. of five independent experiments. Statistical analysis of the significance level of the differences observed between IC_50_ values found for nonactivated and activated DOX was carried out using Student's *t*-test. *P*<0.05 was considered as a significant difference.

## RESULTS

### Reduction of DOX by CPR-spectroscopic studies

**Figure 1:**
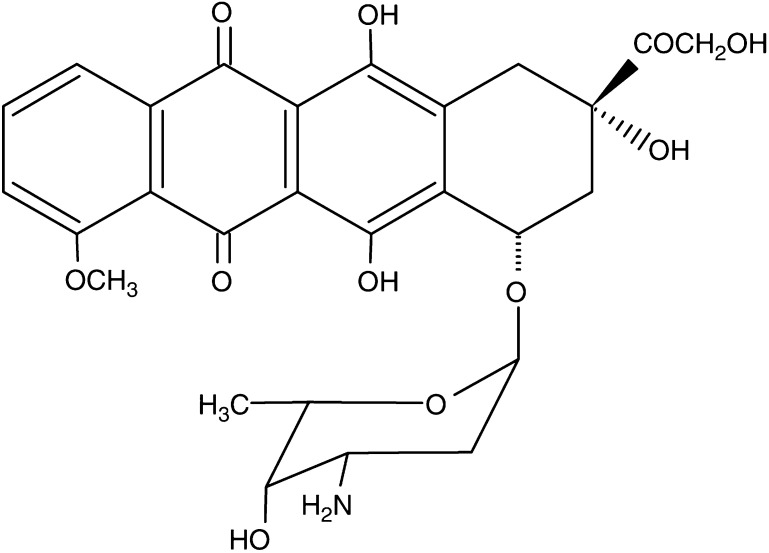
Structure of doxorubicin (DOX).

**Figure 2:**
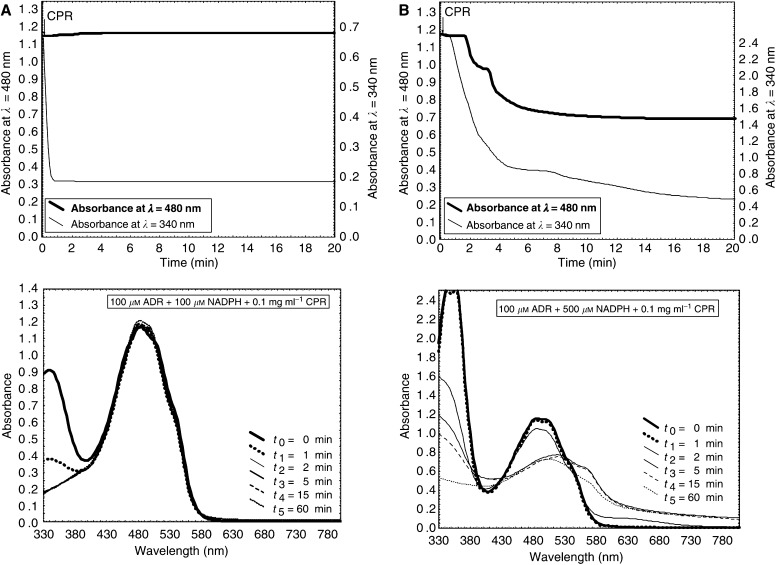

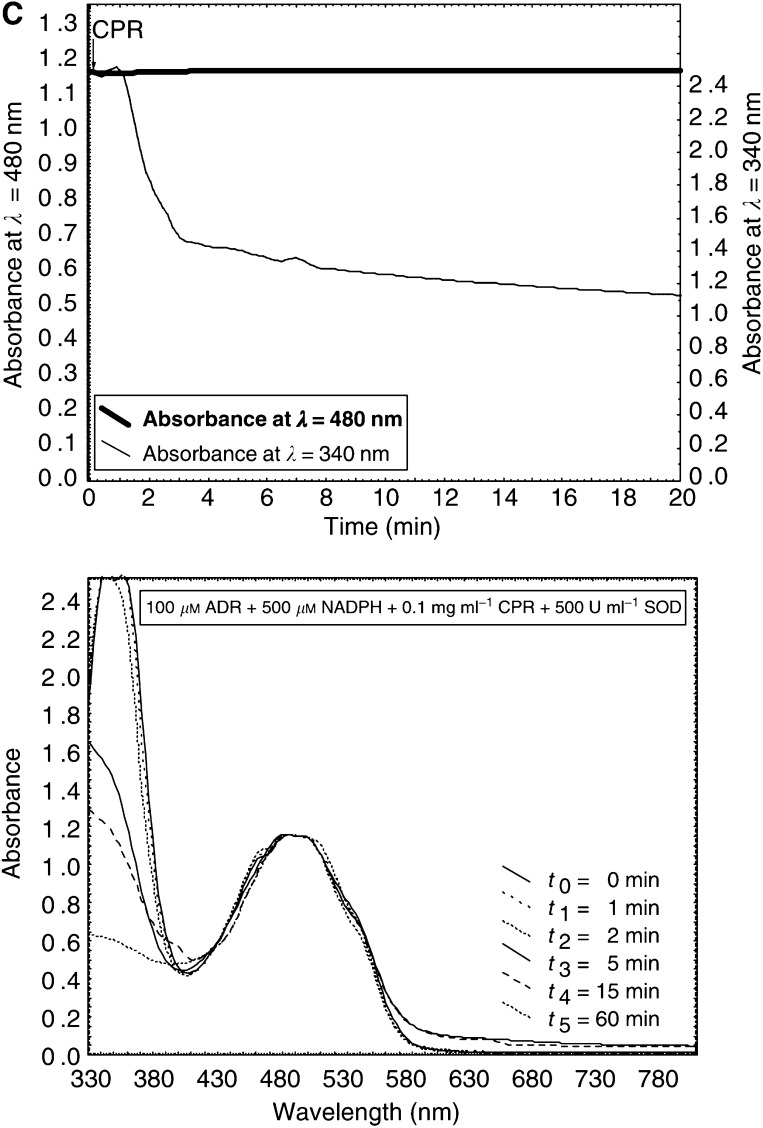
Spectroscopic changes followed during incubation of DOX in enzymatic systems. The selected absorption wavelengths, 340 and 480 nm, represent the maximum absorption wavelengths for NADPH and DOX, respectively. The samples contained the following: (**A**) 100 *μ*M DOX, 100 *μ*M NADPH and 0.1 mg ml^−1^ CPR; (**B**) 100 *μ*M DOX, 500 *μ*M NADPH and 0.1 mg ml^−1^ CPR; (**C**) 100 *μ*M DOX, 500 *μ*M NADPH, 0.1 mg ml^−1^ CPR and 500 U ml^−1^ SOD. The measurements were carried out in 0.01 M K_2_HPO_4_/KH_2_PO_4_ buffer (pH 7.25) at 37°C. The reactions were initiated by the addition of CPR. Data shown are from a representative experiment.

The structure of DOX is presented in Figure 1. The effect of NADPH concentration on the enzymatic reduction of DOX by CPR has been studied in a wide range (100 *μ*M, 250 *μ*M, 300 *μ*M, 350 *μ*M, 500 *μ*M, 1 mM, 2 mM). Figure 2 illustrates the representative absorption spectra of samples recorded during the incubation of 100 *μ*M DOX with 0.1 mg ml^−1^ CPR in the presence of NADPH at low concentration (obtained at 100 *μ*M NADPH) (Figure 2A) or at high concentration (obtained at 500 *μ*M NADPH) (Figure 2B). Figure 2C shows results found for the sample containing 100 *μ*M DOX, 500 *μ*M NADPH, 0.1 mg ml^−1^ CPR and additionally 500 U ml^−1^ SOD. The absorption measurements at selected absorption wavelengths, 340 and 480 nm, were also carried out continuously for each sample studied. These selected wavelengths represent the maximum absorption wavelengths for NADPH (340 nm) and DOX (480 nm).

It was found that at low NADPH concentration lying in the range of 100–350 *μ*M, a very important decrease in the absorption intensity at 340 nm (*A*_340 nm_) was observed immediately after the addition of CPR indicating that DOX caused a high stimulation of NADPH oxidation catalysed by CPR, but no changes were observed in the absorption spectra of DOX up to 3 h (the presented data show the absorption spectra recorded for the first 60 min only). In contrast, at high NADPH concentration (500 *μ*M–2 mM), after the addition of CPR, not only the decrease in *A*_340 nm_ was observed but after about 2 min the important decrease in *A*_480 nm_ was also observed followed by further modifications of absorption spectra of DOX. About 5 min after the addition of CPR, the important shift of the absorption band of DOX was observed indicating the modifications in the chromophore part of the drug. During the prolonged incubation (1 h), the aglycone precipitation occurred. It was also found that after the addition of SOD to the enzymatic samples containing CPR and high concentrations of NADPH (500 *μ*M–2 mM), only the rapid oxidation of NADPH occurred, but no changes were observed in the absorption spectra of DOX (Figure 2C).

### The cytotoxic activity of DOX towards HL60 cell line and its multidrug-resistant sublines: HL60/VINC and HL60/DOX

**Figure 3:**
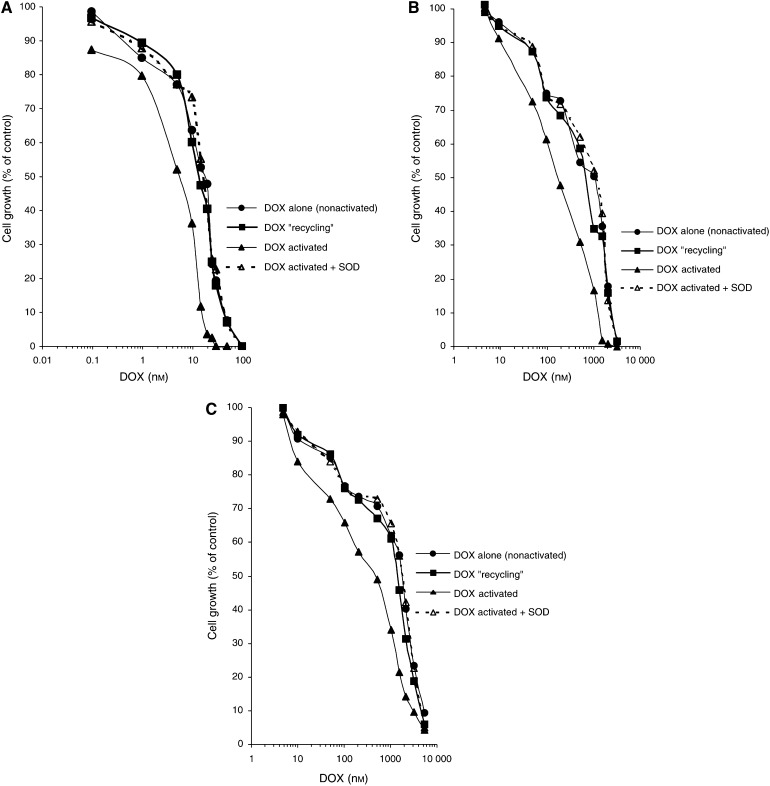
Cytotoxic activity of doxorubicin (DOX) towards (**A**) HL60 cells, (**B**) HL60/VINC cells and (**C**) HL60/DOX cells. The enzymatic samples contained as follows: for DOX ‘recycling’: 100 *μ*M DOX, 100 *μ*M NADPH and 0.1 mg ml^−1^ CPR; for DOX activated (undergoing reductive conversion): 100 *μ*M DOX, 500 *μ*M NADPH and 0.1 mg ml^−1^ CPR; for DOX activated+SOD: 100 *μ*M DOX, 500 *μ*M NADPH, 0.1 mg ml^−1^ CPR and 500 U ml^−1^ SOD (0.01 M K_2_HPO_4_/KH_2_PO_4_ buffer, pH 7.25; 37°C). The appropriate volumes of the enzymatic sample were added directly to the cell suspension to yield the DOX concentration varying in the range of 0.1–100 nM, 5 nM–3 *μ*M and 5 nM–5 *μ*M for HL60 (**A**), HL60/VINC (**B**) and HL60/DOX (**C**) cells, respectively. The cytotoxic effect of DOX was determined by incubating cells (10^5^) with 10 different concentrations of the compound for 72 h. The data points are from a representative experiment.

The ability of DOX to inhibit the growth of human promyelocytic leukaemia HL60 cell line as well as its MDR sublines exhibiting two different phenotypes of MDR related to the overexpression of P-glycoprotein (HL60/VINC) or MRP1 (HL60/DOX) was studied in the presence or in the absence of exogenously added NADPH and CPR. All control cells proliferated during 72 h. The growth rate of the parent cells (HL60) was comparable with both resistant cells used in the study (HL60/VINC and HL60/DOX). All cultures initiated at a density of 10^5^ cells ml^−1^ grew to about 10^6^ cells ml^−1^ (control count) in 72 h. The cytotoxic effect of DOX was determined by incubating cells (10^5^) with 10 different concentrations of DOX for 72 h. In calculating cell growth (% of control), untreated cells were used as the control. The results obtained for each cell line are illustrated in Figure 3. Additional assays carried out for buffer alone, NADPH alone or CPR alone at the highest concentrations used in *in vitro* studies showed that these agents had no effect on cell growth. In all, 100% cell growth of control HL60, HL60/VINC and HL60/DOX was observed at the highest percentage of buffer employed in the culture medium (2%, v v^−1^) as well as at highest concentrations of NADPH (2, 60 and 100 *μ*M in the case of HL60, HL60/VINC and HL60/DOX cells, respectively) or CPR used (0.1, 3 and 5 *μ*g ml^−1^ in the case of HL60, HL60/VINC and HL60/DOX cells, respectively). Control assays carried out for enzymatic samples containing simultaneously NADPH and CPR at the same concentration ranges showed that they have neither any effect on cell growth (100% of control cell growth was also observed, data not presented).

Our results (Figure 3A) showed that the incubation of HL60 cells with DOX pretreated in the presence of CPR and low NADPH concentration (100–350 *μ*M) had no effect in increasing its cytotoxicity in comparison with DOX alone. In contrast, the incubation of HL60 cells with DOX pretreated with CPR at high NADPH concentration (500 *μ*M–2 mM) resulted in an important increase in cell growth inhibition. Higher NADPH concentrations used in enzymatic samples (1, 2 mM) did not result in further increase in DOX cytotoxic activity in comparison to results found employing samples that contained 500 *μ*M NADPH. The increasing effect in cytotoxic acivity of DOX was conserved even when the drug was added 10 min after the preincubation step with NADPH and CPR (data not presented). In contrast, this effect was completely abolished in the presence of 500 U ml^−1^ SOD added to the enzymatic sample.

The same results were found for multidrug-resistant sublines: HL60/VINC and HL60/DOX (Figure 3B and C). The pretreatment of DOX in the presence of CPR and low NADPH concentration (100–350 *μ*M) had no effect in increasing its cytotoxicity against resistant tumour sublines. In contrast, an important increase in the cytotoxic activity of DOX after its pretreatment with CPR at high NADPH concentration (500 *μ*M–2 mM) was observed against HL60/VINC as well as HL60/DOX cells. Similarly to results found for sensitive HL60 cells, higher NADPH concentrations used in enzymatic samples (1, 2 mM) did not result in further increase in DOX cytotoxic activity against both resistant cell lines in comparison to results found employing samples that contained 500 *μ*M NADPH.

IC_50_ values (DOX concentrations required to inhibit 50% of cell growth) are summarised in Table 1. As can be seen, the important decrease in IC_50_ values was observed for DOX activated by CPR in comparison to DOX alone (nonactivated) for both sensitive and resistant cell lines examined. The most important decrease in IC_50_ value was observed in the case of HL60/DOX cells. IC_50_ value found for DOX activated was equal to 571±199 nM *vs* a value of 1644±408 nM found for DOX alone (nonactivated) (*P*<0.0001).

Additional studies performed for vincristine, a nonbioreductive antitumour agent, showed that this compound was not able to stimulate NADPH oxidation in the presence of CPR and no modification in cytotoxic activity of vincristine was observed in the presence of exogenously added CPR against HL60 as well as HL60/VINC and HL60/DOX cells (data not presented).

## DISCUSSION

The ability of neoplastic cells to develop MDR to chemotherapeutic agents (e.g. anthracyclines, vinca alkaloids, podophylotoxins, colchicine) structurally dissimilar and having different intracellular targets constitutes the major problem in cancer therapy (Chaudhary and Roninson, 1993). The MDR transporters (e.g. P-glycoprotein, MRP1) are responsible for the active efflux of drugs out of resistant cells resulting in the decreased intracellular accumulation insufficient to inhibit resistant cell proliferation (Gottesman and Pastan, 1993; Loe *et al*, 1996). It was reported by several researchers that DOX enters into sensitive cells and binds especially to the nucleus (Tarasiuk *et al*, 1989; Tarasiuk and Garnier-Suillerot, 1992; Skladanowski and Konopa, 1994; Taatjes and Koch, 2001; Cutts *et al*, 2003). It has also been presented that in the case of nonactivated DOX, the kinetics of cellular uptake was comparable with the rate of the efflux by MDR exporting pumps (P-glycoprotein and MRP1). Consequently, a drastic decrease in the cellular accumulation of the drug determining its cytotoxic activity was observed (Mankhetkorn *et al*, 1996; Marbeuf-Gueye *et al*, 1998, 1999). Furthermore, there is an increasing number of data demonstrating that the enzymatic activation of DOX results in the formation of reactive intermediates capable of alkylation or crosslinking binding with DNA (Cummings *et al*, 1991; Cullinane *et al*, 1994; Skladanowski and Konopa, 1994). Taking these findings together it would be assumed that oxidoreductases involved in drug metabolic activation could generate reactive intermediates of DOX able to irreversibly bind to DNA before being removed from the resistant cells by MDR exporting pumps. This approach could give a possibility to restore the activity of this bioreductive antitumour agent against multidrug-resistant tumour cells.

In this study, we investigated the role of reductive activation of DOX by the human liver CPR in increasing its cytotoxic activity against human promyelocytic leukaemia cells exhibiting two different phenotypes of MDR related to the overexpression of P-glycoprotein (HL60/VINC) or MRP1 (HL60/DOX). Our previous results showed that the reduction of DOX by CPR and the formation of metabolic intermediates did not occur in the absence of oxygen (Bartoszek *et al*, 1999). However, low oxygen content found often in solid tumours is rather not characteristic for promyelocytic leukaemia studied in the work.

**Figure 4:**
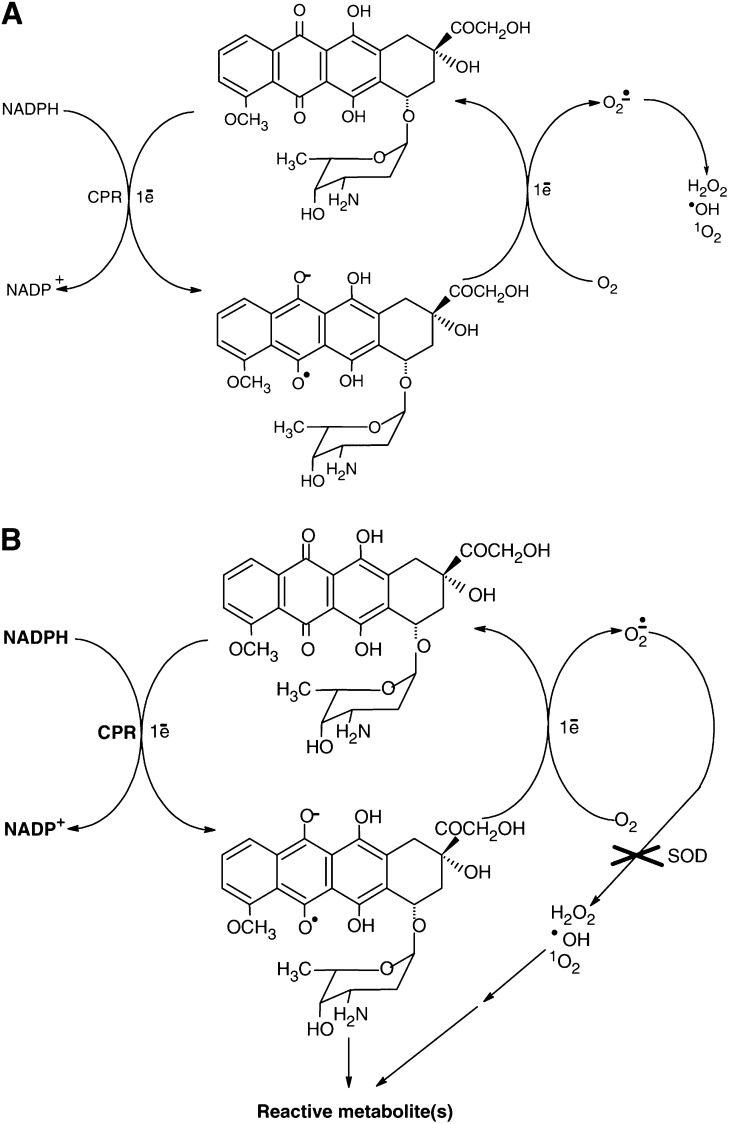
Schema representing the reduction of DOX by CPR: (**A**) redox cycling at low NADPH concentration (100–350 *μ*M); (**B**) reductive conversion at high NADPH concentration (500 *μ*M–2 mM).

Spectroscopic studies performed during incubation of DOX in enzymatic systems containing CPR and various amounts of NADPH showed that the availability of NADPH as a cofactor of enzymatic reactions was a crucial factor determining the route of drug activation. The similar findings were also reported for tirapazamine activation by CPR (Saunders *et al*, 2000). It is proposed that NADPH could participate in forming a coupled interactive system and in consequence constitute a control point in drug activation by cellular oxidoreductases. It was found that at low NADPH concentration lying in the range of 100–350 *μ*M, a high stimulation of NADPH oxidation catalysed by CPR occurred but no changes were observed in the absorption spectra of the drug. It suggests that under these experimental conditions DOX underwent only the redox cycling activation accompanied by the rapid consumption of NADPH. In contrast, at higher NADPH concentration the important shift of the absorption band of DOX was observed indicating the modifications in the chromophore part of the drug. Thus, under these conditions DOX was not only very effective in NADPH stimulation but also concomitantly underwent a multistage chemical transformation upon CPR catalysis. It was found that the minimal concentration of NADPH required for a metabolic activation of DOX occurring with the modification of the chromophore part of the drug was equal to 500 *μ*M. It was also evidenced that the addition of SOD decomposing O_2_^•^ abolished the reductive conversion of DOX by CPR observed in the presence of high concentrations of NADPH (500 *μ*M–2 mM). It indicates that the first stage of DOX reduction by CPR, undergoing reductive activation probably according to the mechanism of the redox cycling, has the key importance for the metabolic conversion of DOX. It seems that O_2_^•^ could be involved (directly or indirectly) in chemical transformations of DOX. However, the exact role of O_2_^•^ in further steps of the metabolic conversion of DOX remains to be clarified. In Figure 4, schema summarising the reduction of DOX by CPR under various experimental conditions studied in the work are presented.

In the second part of the study, it was demonstrated that the presence of CPR catalysing only the redox cycling of DOX had no effect in modulating its cytotoxicity against sensitive HL60 as well as resistant HL60/VINC and HL60/DOX cells in comparison to DOX alone. Consequently, the formation of degradation products of DOX having the same absorption spectra as the parent drug (e.g. aglycone metabolites or doxorubicinol) and the contribution of these products to cytotoxic activity should be rather excluded because it is well known that DOX aglycone has no cytotoxic activity and doxorubicinol was about 20 times less cytotoxic than DOX towards tumour cells (Olson *et al*, 1988; Bernardini *et al*, 1991; Ferrazzi *et al*, 1991). Thus, it seems that the cytotoxic activity observed *in vitro* at low NADPH concentration was caused only by DOX regenerated in the redox cycling. In contrast, after a metabolic conversion of DOX by CPR occurring at high NADPH concentration (at least 500 *μ*M) an important increase in its cytotoxic activity was observed against HL60 as well as HL60/VINC and HL60/DOX cells. It should be rather excluded that NADPH or CPR added exogenously could get into the cell. On the basis of the results obtained with the aid of exogenous SOD (not being able to penetrate across the plasma membrane) that abolished completely the increasing effect in cytotoxic activity of DOX observed in the same experimental conditions in the absence of SOD, it should be rather proposed that DOX is activated outside the cells by extracellularly added CPR. It is worth noting that the data presented in the study (similar increase of about 2–3-fold in the cytotoxic activity of DOX against resistant HL60/VINC and HL60/DOX cells as it was observed in the case of sensitive HL60 cell line) suggest that this (these) metabolite(s) generated extracellulary is (are) able to enter the cell and bind to cellular targets before being extruded by MDR exporting pumps. At this stage of our research, the nature of this (these) metabolite(s) is unknown. It is evident that its (their) formation is related to the modifications of the chromophore part of DOX resulting in the important changes of its absorption spectrum. The data obtained also evidenced that it was (they were) (a) relatively long-lived specie(s) because the increasing effect in cytotoxic activity of DOX was conserved even when the drug was added 10 min after the preincubation step with NADPH and CPR. Identification of this (these) reactive metabolite(s) as well as investigating its (their) rate of cellular accumulation, level of nuclear retention and rate of active export by MDR exporting pumps (P-glycoprotein, MRP1) (Tarasiuk *et al*, 1989, 2004; Tkaczyk-Gobis *et al*, 2001) is needed in order to clarify the role of reductive activation of DOX by cellular oxidoreductases in the mode of action of this drug in regard to resistant tumour cells. These studies are in progress in our laboratory.

Presented data showed that the reductive activation of DOX by exogenously added CPR resulted in the 2- to 3-fold increase in cytotoxic activity of the drug (IC_50_ value were decreased from 18.6±8.9 to 9.3±4.2 nM, from 433±133 to 256±68 nM and from 1644±408 to 571±197 nM in the case of HL60, HL60/VINC and HL60/DOX, respectively), whereas HL60/VINC and HL60/DOX cells were, respectively, 23- and 88-fold resistant compared to the sensitive cells. However, taking into account that the plasma level of DOX achievable clinically is in the range of 1–2 *μ*M as well as that high drug concentrations could be responsible for severe dose-dependent toxic side effects, it seems that the reported decreases in IC_50_ values found for resistant cell lines (HL60/VINC and HL60/DOX) could be of clinical importance for the treatment of tumours resistant to classical chemotherapy. Having in mind literature data, it was not surprising that the formation of reactive intermediates of DOX by exogenous CPR resulted in increasing its cytotoxic activity against sensitive HL60 cells. The obtained results are in agreement with data previously reported for human breast tumour MCF-7 cells (Bartoszek and Wolf, 1992). However, it seems that the importance of the presented data concerns the demonstration that the potentiation of DOX activity would also be obtained in resistant cells overexpressing MDR exporting pumps able to extrude drugs from the cell. Thus, it would be proposed that this (these) reactive metabolite(s) generated by CPR is (are) able to bind to cellular targets before being pumped out of the cell by P-glycoprotein or MRP1. Furthermore, based on presented results, it seems that the enhancement in cytotoxic activity of DOX in the presence of exogenously added CPR and NADPH did not involve additionally other specific resistance mechanisms.

According to the literature data, metabolic activation of drugs can also undergo efficiently inside tumour cells. It is known that intracellular CPR expression involved in the activation of bioreductive agents can be modulated in cells by many internal factors such as oxygen deficiency, intracellular pH changes and by malignant transformations themselves (Adams and Stratford, 1994; Forkert *et al*, 1996). The development of an enzyme-directed gene therapy (GDEPT) approach targeted toward specific reductase enzyme for antitumour drug bioactivation is also in progress in many laboratories (Cowen *et al*, 2003). However, literature data reported for some tumour cells overexpressing CPR showed the lack of enhanced sensitivity of these cells to the cytotoxicity of DOX (Schmalix *et al*, 1996; Ramji *et al*, 2003), whereas a study by Niitsu *et al* (2000) reported that decrease in intracellular CPR level increased DOX cytotoxicity. It suggests that the reductive activation of DOX *in situ* in target cells by CPR could be influenced by several cellular factors, for example, bioavailability of NADPH, competitive metabolic pathways of the drug catalysed by other enzymes depending on their cellular levels or rapid decomposition of ROS and DOX free radicals by the antioxidant defence system of the cell. In the presented study, the metabolic activation of DOX occurred under well-controlled experimental conditions and the obtained results indicate that the reactive metabolite(s) formed extracellularly is (are) able to penetrate into the cell and bind to intracellular targets before being decomposed. Nevertheless, in further steps of the study it would be interesting to also examine the intracellular activation of DOX in leukaemia cells overexpressing CPR.

## CONCLUSION

The data presented in the study suggest that the reductive activation of DOX by exogenously added CPR constitutes a possibility to potentiate the activity of this clinically important drug against multidrug-resistant leukaemia cells. Having in mind the relative stability of reactive intermediate(s) generated extracellulary and its (their) ability to increase the cytotoxic activity of the drug, the metabolic activation of DOX by exogenous CPR just before being administrated might be perhaps taken into consideration. However, the clinical significance of the presented results obtained in the model system remains to be elucidated. Therefore, further studies are needed to prove the potential importance of the presented data in leukaemia therapy.

## Figures and Tables

**Table 1:** Cytotoxic activity of doxorubicin towards HL60 cell line and its multidrug resistant sublines: HL60/VINC and HL60/DOX

	**IC_50_ (nM)**	
**Cell line**	**Doxorubicin nonactivated**	**Doxorubicin activated**
HL60	18.6±8.9	9.3±4.2
HL60/VINC	438±133	256±68^a^
HL60/DOX	1644±408	571±197^b^

IC_50_ is the doxorubicin (DOX) concentration required to inhibit 50% of cell growth. The values represent mean±s.d. of five independent experiments. The significance level of the differences observed (Students's *t*-test).

a *P*<0.05;

b *P*<0.0001 *vs* values found for DOX alone (nonactivated).
